# Development and Evaluation of a Mentor Handbook to Support Accelerated Pathway to Medical Education (APME) Students

**DOI:** 10.7759/cureus.101062

**Published:** 2026-01-08

**Authors:** Chanel Ross, Lori Zarmer, Dylan Joule, Chloe Eckert, Tejal Parikh

**Affiliations:** 1 College of Medicine, University of Arizona College of Medicine - Tucson, Tucson, USA; 2 Family Medicine, University of Arizona College of Medicine - Tucson, Tucson, USA

**Keywords:** academic and wellness support, accelerated bs/md program, accelerated pathway to medical education (apme), medical education, medical student mentorship, mentorship handbook development, peer networking in medical education, student resource evaluation

## Abstract

Introduction: Combined baccalaureate-MD programs offer an accelerated pathway to medical education, yet mentor-facing guidance and evaluated support tools for these programs remain limited. The University of Arizona College of Medicine-Tucson established the Accelerated Pathway to Medical Education (APME) program in 2021, alongside a near-peer mentorship initiative connecting current medical students with APME participants. To address the absence of structured, mentor-facing resources tailored to accelerated tracks, we developed and evaluated a mentor handbook designed to support academic navigation, professional development, wellness, and peer connectivity for APME students, with evaluation focused on student perceptions rather than observed behavioral outcomes.

Materials and methods: This cross-sectional, descriptive pilot study involved the development, dissemination, and perceptual evaluation of a digital mentor handbook. Content was created through review of institutional resources, consultations with program leaders and upperclassmen, and input from current mentors. Following dissemination, an anonymous electronic questionnaire was distributed to all eligible APME students and the new APME mentors (N=14) during the 2024-2025 academic year; 10 participants responded (71% response rate). Quantitative data from 5-point Likert scale questions assessing perceived clarity, relevance, and usefulness were summarized using frequencies, percentages, and p-values, while qualitative responses underwent reflexive thematic analysis by two independent coders.

Results: Respondents reported high perceived clarity (n=10/10 satisfied or very satisfied; p=0.001), relevance (n=8/10, p=0.055), and helpfulness (n=8/10; p=0.055). The most frequently selected useful sections were Mentor Contact Information (n=8/10), followed by the Cold Email Template (n=5/10). Qualitative feedback highlighted the value of peer contact information for fostering connections but raised concerns that including recommended course lists and early introduction of study tools could create unnecessary pressure or information overload. Students also recommended adding a section for on- and off-campus study locations.

Conclusion: In this exploratory pilot study with a small sample size, the mentor handbook was perceived as a clear and relevant support tool for an accelerated medical pathway. Findings reflect subjective perceptions rather than demonstrated behavioral or academic outcomes and should be interpreted accordingly. Future work, including longitudinal follow-up and multi-institutional evaluation, will be critical to strengthen the evidence base, assess sustained impact, and determine generalizability across programs. Ongoing, student-driven revisions and longitudinal evaluations will be essential to optimize relevance, mitigate bias, and assess broader impact across future cohorts.

## Introduction

Currently, there are 45 combined Baccalaureate-MD programs in the United States, with total training duration ranging from six to nine years. While these programs may reduce time and cost to degree, they also introduce unique academic, psychosocial, and professional identity challenges, including early career decision-making, intensified academic pacing, and reduced time for exploratory development [1]. Despite the growth of such programs, most mentorship models applied to accelerated pathways mirror those used in traditional premedical or medical education and rely heavily on informal advising relationships, and there remains a paucity of evidence-based, mentor-facing resources designed specifically to support students navigating these accelerated pathways.

Current literature suggests that accelerated pathway students perform comparably to traditional medical students on quantitative measures [2,3]. It is unclear if this reflects the strength of curricular structure or individual study habits, although there is some evidence that students in programs that required liberal arts courses had lower self-perceived preparedness [4]. It is harder to quantify competency in interpersonal skills and leadership, which are both major components of clinical practice. Interviews with combined program graduates who had completed medical school at least 15 years ago revealed the importance of strong role models and informal leadership opportunities for professional development [5].

The Accelerated Pathway to Medical Education (APME) program at the University of Arizona-Tucson, created in 2021, is a seven-year combined track, with its inaugural class set to graduate in 2028. The curriculum condenses the pre-medical phase into three years, followed by the traditional four years of medical school. Unlike traditional premedical pathways, APME students commit to a medical career early, often before exposure to common advising structures available to traditional applicants. Mentors, typically near-peer medical students, are therefore tasked with guiding students through academic planning, extracurricular engagement, wellness, and professional development in a compressed timeframe, often without standardized tools to ensure consistency, accuracy, or alignment with program goals.

Mentorship has been associated with improved academic engagement, career development, and wellness in medical education [6]. Frameworks such as near-peer mentoring [7], social cognitive theory [8], and community of practice [9] emphasize role modeling, belonging, and scaffolding of professional identity formation. However, existing mentorship interventions primarily extend these frameworks through relational or programmatic structures (e.g., advisor matching, workshops, or faculty-led initiatives) rather than through evaluated, reusable mentor-facing tools. While one study highlighted the critical role of mentorship in supporting Baccalaureate-MD students, reporting that strong advisor relationships and participation in structured programs were key factors promoting student engagement in research [10], few studies examine how mentors are supported with standardized resources or evaluate tools designed to promote consistency while allowing flexibility for individual mentoring relationships.

To address this gap, we developed a mentor handbook as a novel, mentor-facing intervention that complements, rather than replaces, existing mentorship relationships. Unlike traditional mentorship models that depend on individual mentor experience or time-limited training sessions, this handbook was designed to provide a centralized, scalable, and updatable resource that standardizes essential guidance while allowing mentors and students to engage with content asynchronously and as needed. This approach represents a shift from extending mentorship structures alone to explicitly supporting mentors through a theory-informed, evaluable resource.

This study aimed to (1) develop a mentor handbook grounded in mentorship theory and local needs to support APME students and mentors; (2) evaluate perceived clarity, relevance, and usefulness of the handbook among APME students and mentors; and (3) identify specific areas for improvement to inform iterative revisions. We aim for the resources in this handbook to contribute to the success of APME students and to be continuously updated and expanded in the coming years, providing ongoing support for future cohorts. This pilot evaluation focuses on perceived usefulness rather than objective academic or behavioral outcomes.

## Materials and methods

Study design and setting

This was a cross-sectional, descriptive pilot study conducted at the University of Arizona College of Medicine - Tucson during the 2024-2025 academic year. As a pilot investigation, the study was designed to assess feasibility and perceived usefulness rather than to support definitive statistical inference. The very small sample size inherently limits statistical power, precision, and generalizability of findings.

Needs assessment and handbook development

Handbook content was informed by an informal needs analysis, including (1) review of institutional advising materials, (2) consultation with APME program leadership, and (3) structured input from current mentors and APME students regarding common challenges and frequently asked questions. Content domains were guided by near-peer mentoring principles and self-determination theory, emphasizing autonomy, competence, and relatedness. The content domains included mentor contact information, academic advising (study habits, recommended undergraduate courses and academic resources), how to get involved in extracurricular activities (research, clinical experience/shadowing, volunteering, leadership), a cold email template for reaching out to other professionals, personal and professional development resources, and wellness resources (mental health and lifestyle skills). The handbook was reviewed by faculty and program administrators prior to distribution. The handbook was disseminated in digital format (PDF) to enhance accessibility and reproducibility.

Questionnaire development

Following the dissemination of the mentor handbook, an anonymous electronic questionnaire was created using Google Forms (Google LLC, Mountain View, CA, USA) to assess students’ perceptions of its usefulness. The questionnaire included both quantitative and qualitative items: 5-point Likert scale questions evaluating the handbook’s clarity, relevance, and helpfulness, a multiple-response (“select all that apply”) question identifying the most useful sections, and an open-ended question allowing students to provide feedback and suggestions for improvement (Table 1). Items were reviewed by faculty advisors for face validity and clarity prior to distribution. The instrument was not formally validated, consistent with its intended use as a quality-improvement pilot tool, and responses may be subject to social desirability bias given participants’ affiliation with the program.

**Table 1:** APME mentor handbook satisfaction and feedback survey. Survey items assessed mentor perceptions of the handbook across three domains - helpfulness, relevance, and clarity - using a 5-point Likert scale (very dissatisfied to very satisfied). Participants also indicated which sections were most useful (multiple selections allowed) and provided open-ended feedback on suggested additions or removals. APME: Accelerated Pathway to Medical Education

Domain	Survey Item	Response Options
Helpfulness	How satisfied are you with the helpfulness of the material included in the mentor handbook?	Very satisfied; Satisfied; Neutral; Dissatisfied; Very dissatisfied
Relevance	How satisfied are you with the relevance of the material included in the mentor handbook?	Very satisfied; Satisfied; Neutral; Dissatisfied; Very dissatisfied
Clarity	How satisfied are you with the clarity of the writing in the mentor handbook?	Very satisfied; Satisfied; Neutral; Dissatisfied; Very dissatisfied
Content Utility	Which sections of the mentor handbook were the most useful? (Select all that apply)	Mentor Contact Information; Academics; Extracurriculars; Cold Email Template; Personal and Professional Development; Mental Health and Wellbeing; Other
Open Feedback	What information would be helpful to add or remove from the handbook?	Free-text response

Questionnaire distribution

The survey was distributed via email using Google Forms to all nine students enrolled in the APME program and the five new APME mentors during the 2024-2025 academic year. The sample size of 14 was determined by the total number of students and mentors in the program, as these were the only individuals eligible to participate. Responses were collected over a one-week period. Of the 14 individuals invited, 10 completed the survey, yielding a 71% response rate. This short evaluation window limits assessment of sustained engagement with the handbook or longer-term impact on mentoring experiences.

Data analysis

Quantitative data from the questionnaire were analyzed in Google Sheets (Google LLC) using frequencies and percentages, with raw counts reported alongside percentages. Given the nature of Likert-scale data and the very small sample size, inferential analyses using two-sided t-tests were conducted in an exploratory manner only to compare observed positive responses (very satisfied and satisfied) against non-positive responses (neutral, dissatisfied, very dissatisfied) at p=0.50, recognizing that statistical interpretation is limited and results should be considered hypothesis-generating rather than confirmatory. The qualitative responses from the open-ended questions were reviewed using reflexive thematic analysis following Braun and Clarke’s six-step approach [11]. Two investigators independently coded responses, reconciled discrepancies through discussion, and agreed on final themes. No qualitative data analysis software was used.

Ethical considerations

The University of Arizona Institutional Review Board reviewed this project and determined it to be exempt as an educational quality improvement initiative. Informed consent was implied by voluntary participation; no identifying information was collected. Data was stored on password-protected institutional systems.

## Results

Of the 10 respondents, clarity was rated positively (satisfied or very satisfied) by all participants (n=10/10). This uniform direction of response indicates strong perceived clarity, although statistical significance should be interpreted cautiously given the small sample size and exploratory nature of the analysis. Relevance and helpfulness were each rated positively by eight of 10 respondents, demonstrating a consistent positive trend across domains rather than definitive statistical effects. While exploratory p-values are reported for transparency, interpretation is guided primarily by response direction and consistency across items. Table 2 reports raw counts, percentages, and corresponding p-values for all Likert-scale items.

**Table 2:** Positive responses were defined as very satisfied or satisfied. P-values were generated from exploratory two-sided t-tests and are presented descriptively; emphasis is placed on effect direction and consistency rather than statistical thresholds.

Domain Assessed	Positive Responses (n/N, %)	P-value
Helpfulness of Material	8/10 (80)	0.055
Relevance of Material	8/10 (80)	0.055
Clarity of Writing	10/10 (100)	0.001

**Figure 1:**
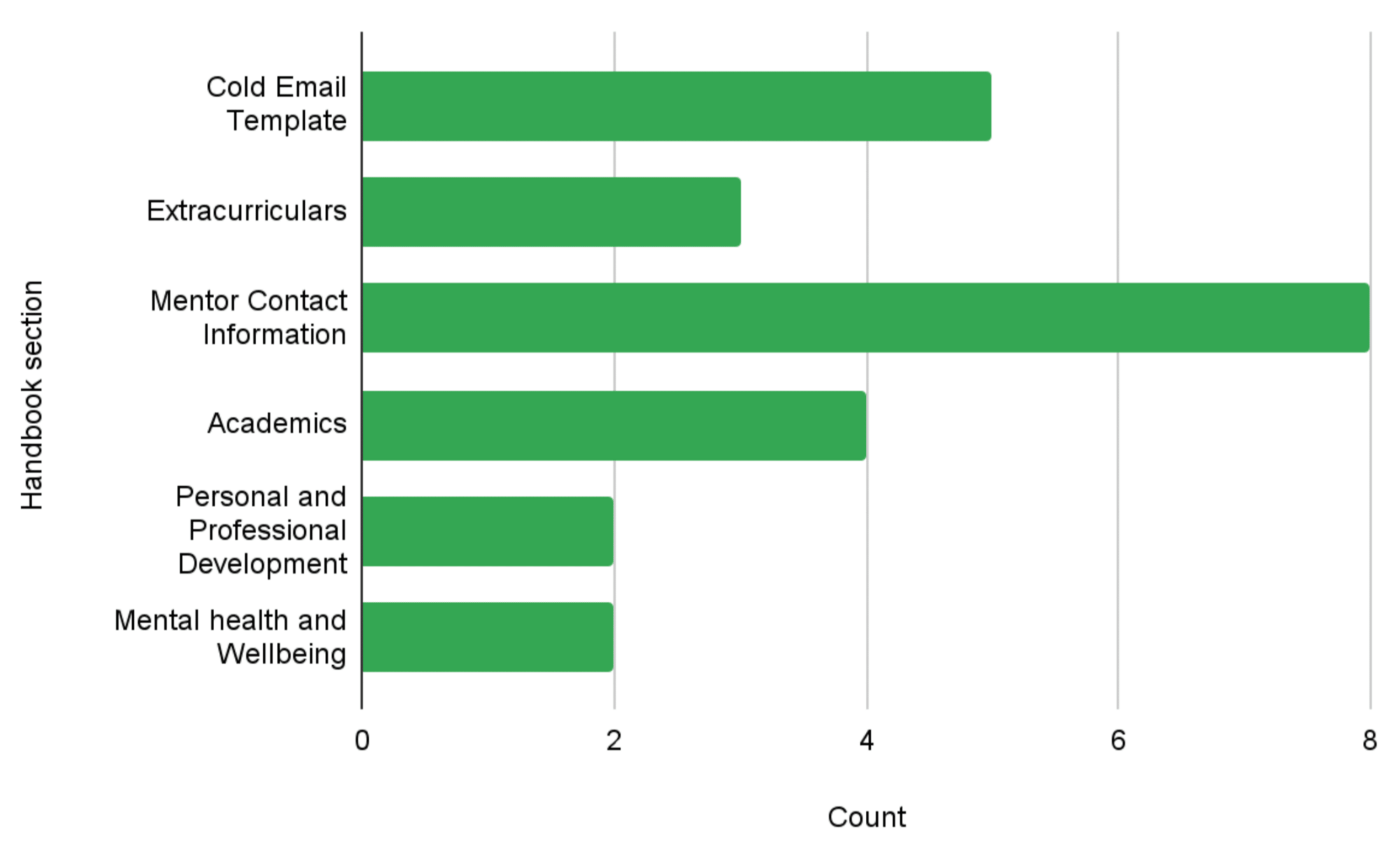
Participant responses to the question, “What sections were the most useful?” Responses are shown as counts for each section of the mentor handbook.

When asked which sections of the handbook were most useful (Figure 1), participants most frequently selected Mentor Contact Information (n=8/10), followed by the Cold Email Template (n=5/10), Academics (n=4/10), and Extracurriculars (n=3/10). Personal and Professional Development and Mental Health and Wellbeing were each selected twice. The ranking of these sections demonstrates convergence between quantitative trends and qualitative feedback regarding practical utility and peer connectivity.

Qualitative feedback further reinforced these patterns (Table 3), highlighting thematic consistency across responses. Participants identified that including the contact information for other APME students would be a particularly useful feature, facilitating easier peer-to-peer outreach and networking. One respondent expressed concerns regarding the inclusion of certain academic recommendations. Specifically, they felt that providing a list of suggested undergraduate courses could be overwhelming for new students, particularly those who are already academically driven. The respondent noted that this content might unintentionally create pressure to follow a specific curriculum, despite these courses having minimal impact on long-term medical school preparedness. They suggested that course selection should be left to individual preference and that students would likely learn about such options organically through peers and faculty. Similarly, the inclusion of Anki (ANKI, San Francisco, CA, USA) was questioned, with feedback indicating that introducing this study tool too early could contribute to information overload. Respondents felt that exposure to such resources should, again, occur naturally over time rather than being mandated or emphasized at the outset of the program. Finally, participants recommended adding a section for on- and off-campus study spots to provide students with practical, location-based resources to complete their academic work.

**Table 3:** Themes, subthemes, and representative participant quotes identified through thematic analysis of open-ended questionnaire responses. APME: Accelerated Pathway to Medical Education

Theme	Subtheme	Representative Quote
Peer Support and Connection	Access to contact information	“Contact information of other APME students make reaching out easier.”
Academic Guidance	Concerns about recommended course lists	“Providing a list of recommended classes may seem overwhelming…they should feel like they can take whatever classes they want.”
	Autonomy in academic decisions	“I don’t think it needs to be brought up…students should determine if this is a resource they want to have.”
Study Tools and Resources	Timing of introducing study aids (e.g., Anki)	“You might not want to include Anki because… it might just feel like a lot thrown at them all at once.”
Wellness and Balance	Campus and community resources	“I think on and off campus study spots would be a great addition to the book!”

## Discussion

The uniformly positive ratings for handbook clarity, supported by a statistically significant t-test, suggest that the handbook’s organization and language were particularly effective. Perceptions of relevance and helpfulness also demonstrated statistical significance, but these values may be underpowered due to the small sample size. These findings reflect participants’ perceptions of usefulness rather than measured educational outcomes, and should be interpreted as preliminary signals within this pilot cohort. These results align with prior literature demonstrating the importance of structured mentorship and accessible resources in combined baccalaureate-MD programs [10], where early guidance can help students navigate the accelerated and intensive curriculum.

The most frequently selected useful component was the Mentor Contact Information section, reflecting the importance of facilitating peer-to-peer connections and network-building within the program. The prominence of peer contact information underscores the central role of relatedness and near-peer support in compressed training environments, aligning with community of practice models [9]. While this suggests potential benefit for cohort integration, we acknowledge that broader programmatic impact remains untested and should not be inferred from these pilot data. This finding supports previous research highlighting strong interpersonal relationships as a key factor in student success and professional development in combined-degree tracks [5]. Providing structured opportunities for these connections will help foster a sense of community and support among cohorts, which is particularly valuable in accelerated pathways where academic and social integration occur on a compressed timeline.

Despite the overall positive reception, participants offered constructive feedback that will help inform future revisions. One respondent expressed concern that the inclusion of a recommended undergraduate course list could unintentionally create pressure on students to follow a specific academic track. These concerns about academic prescriptiveness emphasize the importance of autonomy-supportive design, consistent with self-determination theory [12]. This perception is also consistent with previous findings that highly motivated students in accelerated programs may experience increased stress when faced with perceived academic expectations beyond the formal curriculum [4]. Participants recommended shifting toward a more flexible approach to academic planning, allowing students to explore courses without feeling obligated to adhere to a list.

Similarly, the early introduction of Anki as a study tool was questioned, with some students indicating that such resources may be better if they are introduced later, after students have developed their own learning strategies. This reflects a broader theme in the feedback that students value autonomy in determining when and how to engage with certain academic resources [12]. Overloading first-year undergraduate students with optional yet advanced tools may inadvertently contribute to information fatigue, especially in the context of a new and rigorous program. Participants also suggested adding a section for on- and off-campus study locations, emphasizing the value of including practical, location-based resources in addition to academic and professional development guidance. This recommendation highlights the variable nature of student needs in accelerated programs, where academic success is intertwined with access to conducive learning environments. These recommendations illustrate perceived usefulness for students in this cohort rather than proven outcomes on performance, engagement, or long-term success.

Importantly, all of these findings reflect subjective perceptions rather than objective outcomes. Positive responses may be influenced by social desirability bias within a small, close-knit program. The short interval between handbook distribution and evaluation likely captured initial impressions rather than sustained utility.

Future work should include longitudinal, multi-institutional studies assessing behavioral and academic outcomes, comparative designs evaluating different mentorship modalities, and formal validation of evaluation instruments. While our pilot results suggest potential utility of handbook-based mentorship resources, broader generalization or programmatic reform should be approached cautiously until such evidence is available.

This study had several limitations. First, the sample size was inherently small, as it was limited to the total number of students and mentors in the APME program. As a result, findings may not be generalizable beyond this specific program. Second, responses were self-reported and may be subject to response bias, particularly given the small, close-knit nature of the program. Third, qualitative data were analyzed by study investigators without the use of specialized software, which may introduce subjectivity despite efforts to reach consensus. Fourth, as this was a cross-sectional evaluation conducted shortly after handbook distribution, longer-term effects on student and mentor experiences were not assessed. Lastly, although exploratory p-values were calculated, the study was not powered for inferential testing, and these results should be interpreted as descriptive signals rather than confirmatory evidence. Future studies with larger sample sizes, independent coding of qualitative data, and longitudinal follow-up would strengthen the evidence base for the effectiveness of structured mentorship resources in accelerated medical pathways. Until such studies are conducted, claims regarding broader applicability or programmatic impact should be considered provisional and based on perceived usefulness rather than demonstrated outcomes.

## Conclusions

In this exploratory pilot study with a small sample size, the mentor handbook was perceived as a clear and relevant support tool for an accelerated medical pathway. Findings reflect subjective perceptions rather than demonstrated behavioral or academic outcomes and should be interpreted accordingly. Future work, including longitudinal follow-up and multi-institutional evaluation, will be critical to strengthen the evidence base, assess sustained impact, and determine generalizability across programs. Ongoing, student-driven revisions and longitudinal evaluations will be essential to optimize relevance, mitigate bias, and assess broader impact across future cohorts.
